# Supplementary material for: Are carbon emissions trading and green financial instruments synergistic? -Comprehensive quantitative research based on content analysis
Source: PLoS One. 2024 Mar 7;19(3):e0298601. doi: 10.1371/journal.pone.0298601 (PMC10919601; doi:10.1371/journal.pone.0298601)
Supplement: S1 Data — (ZIP) [file pone.0298601.s001.zip › Data/地级市Z_F工作报告.zip!/╔╜╢1⁄2/╚╒╒╒╩╨/└┤╫╘╣1⁄2╓┌║┼í╛┬φ┐╦╩2╛▌═°í┐_2006.docx]

各位代表：

现在，我代表市人民政府，向大会作政府工作报告，请予审议，并请市政协各位委员和其他列席会议的同志提出意见。

一、“十五”时期政府工作的回顾

新世纪的头五年，是极不平凡的五年。五年来，全市各级政府在省委、省政府和市委的正确领导下，在历届市领导班子打下的良好基础上，团结带领全市人民，高举邓小平理论和“三个代表”重要思想伟大旗帜，认真贯彻落实党的十六大和十六届三中、四中、五中全会精神，解放思想，抢抓机遇，干事创业，坚定不移地推进跨越发展，提前一年完成了“十五”计划确定的各项任务，开创了我市改革开放和现代化建设的新局面。

国民经济持续快速增长，综合实力跃上新台阶。一是发展速度明显加快。“十五”期间我市经济发展速度逐年加快，特别是近年来我们的发展速度、发展后劲、发展活力都走在了全省的前列。初步核算，2005年实现生产总值470亿元（按原核算办法），年均增长15.2%，分别比“九五”和全省平均水平高出2.9和2.2个百分点；人均生产总值16720元，比2000年翻了一番。地方财政收入14.75亿元，按可比口径年均增长23.1%。二是经济结构明显优化。三次产业比例由“九五”末的24.3:39.1:36.6调整为14:53:33，二三产业比重五年上升了10个百分点。农业和农村经济全面发展，粮食生产稳定增长，特色农业成为农民增收的重要产业，林牧渔业规模经营水平不断提高，农业产业化、标准化进程加快，全市销售收入过亿元的农业龙头企业由“九五”末的8家发展到20家。工业呈现出总量扩大、结构优化、效益不断提高的发展势头。规模以上工业增加值157亿元，是“九五”末的5.5倍；销售收入525.5亿元，是“九五”末的6倍；高新技术产业产值增长7.8倍。商贸流通繁荣活跃，银座商城、利群商厦、日百新玛特等一批大型商场相继建成营业，连锁经营、“超市下乡”发展迅速，社会消费品零售总额年均增长14.9%。三是发展后劲明显增强。五年累计完成全社会固定资产投资717.6亿元，是“九五”期间的2.6倍，其中工业投入226亿元，完成投资过亿元的项目54个、过千万元的483个。全市规模以上工业企业发展到485家，五年新增302家；销售收入过亿元的企业72家，新增49家，其中日照钢铁、五征集团分别突破80亿元和50亿元。

港口发展取得重大突破，特色经济成为新亮点。一是港口经济迅猛崛起。顺利完成了日照、岚山两港联合重组，修订完善了港口总体规划，确立了建设两个亿吨大港的目标。五年累计完成港口投资70亿元，是“九五”期间的9.7倍，先后建成矿石、集装箱等38项港口工程，新增吞吐能力4700万吨。2005年港口货物吞吐量达到8421万吨，当年增幅居全国第一、增量居全省第一，是2000年的2.7倍，跨入全国沿海十大港口行列。依托港口优势，开工建设临港工业项目57个，完成投资100.5亿元，日照钢铁等一批大项目先后建成投产。去年临港工业实现销售收入273亿元，比上年翻了一番，占规模以上工业销售收入的52%。港口经济的“龙头”带动作用日益突出。二是大学经济蓬勃发展。先后争引山东体育学院、曲阜师范大学等省内外6所高校到日照投资办学，结束了日照没有大学的历史。大学科技园累计完成投资25亿元、建筑面积160万平方米，在校大学生达到5万人，科研院所达到5家。三是体育经济方兴未艾。成功地承办了国际欧洲级帆船世界锦标赛、世界帆板精英赛等35项国内外重大体育赛事。建成了世界一流的世帆赛基地，并被确定为“国家水上运动训练基地”，加强了奥林匹克水上公园规划建设，为把我市打造成为“水上运动之都”迈出了坚实的步伐。四是旅游经济快速发展。旅游人数和旅游收入年均增长16.8%和20.8%，特别是“渔家乐”民俗游发展到16个村、5万张床位，有6个村被国家旅游局命名为“全国农业旅游示范点”。与此同时，房地产业呈现出勃勃生机，房地产开发投资和商品房销售额年均增长63.7%和65.3%，成为重要的新兴产业。会展经济快速发育，成功举办了三届茶博会、房博会和中韩投资贸易洽谈会，扩大了对外影响，提升了城市形象。

各项改革深入推进，对外开放实现新跨越。以税费改革为重点的农村改革不断深化，通过降低农业税税率和取消除烟叶外的农业特产税，累计为全市农民减轻负担4.1亿元。以建立现代企业制度为目标的企业改革扎实推进，国有中小企业产权制度改革任务基本完成，乡及乡以上企业改制面达到98%，民营担保机构建设实现新突破，非公有制经济蓬勃发展。以市场化为取向的各项改革取得突破性进展，税收属地征管、国库集中支付和粮食、商贸流通体制等改革都取得了明显成效。以转变政府职能、优化发展环境为重点的政府管理体制改革深入推进，设立了行政服务中心，清理削减审批事项67项，行政效能和服务管理水平明显提高。对外开放力度加大，开通了至韩国平泽的客箱班轮，与国外4个城市新建了友城关系。五年累计实际利用外资6.15亿美元。2005年完成进出口总值25.5亿美元，年均增长35.5%。“接轨青岛、融入半岛”成效明显，与青岛及半岛城市的经贸、信息、技术、人才、旅游等合作全面拓展。

基础设施建设力度加大，城市化进程开创新局面。胶新铁路、同三高速公路等一批重大项目相继建成，滨海大道开工建设。电力装机容量达到86万千瓦，全社会用电量比2000年增长2.4倍。邮政、通信事业快速发展，固话网和移动电话用户分别是2000年的2.8倍和7倍，互联网用户从无到有发展到近8万户。开工建设了日照、青峰岭等4座大中型水库除险加固和城市调水供水、沿海防潮堤建设等重点水利工程。城市规划工作进一步加强，开展了城市发展战略研究和新一轮城市总体规划修编，确立了“双城一区、轴向拓展”的带形城市空间发展战略，详规覆盖率比“九五”末提高54个百分点。城市建设步伐明显加快，五年累计完成城建投资63.2亿元，是“九五”时期的10.5倍，城市化水平达到42%，比“九五”末提高6.6个百分点。先后建成了万平口生态广场、灯塔广场、迎宾路、山海路等一批精品工程，成为展示日照对外形象的重要窗口。城市管理水平明显提高，加强了城市综合执法管理，开展了大规模的城市环境综合整治活动，城市绿化、美化、亮化水平上了一个新台阶。五年新增城市道路220公里，人均公共绿地由“九五”末的10.5平方米增加到19平方米。先后荣获“中国优秀旅游城市”、“国家环境保护模范城市”、“创建国家循环经济示范市”、“国家园林城市”和“山东人居环境奖”等荣誉称号，创建“国家卫生城市”已顺利通过技术核查。两县两区城镇建设得到加强，城乡面貌发生了深刻变化。

社会事业全面进步，人民生活有了新改善。“科教兴市”战略深入实施。五年共取得重要科技成果463项，专利申请量是“九五”期间的4.2倍。新创山东名牌23个，“三维”桑茧生丝和“雪青”绿茶分别荣获“中国名牌产品”和“中国驰名商标”称号，实现了我市无中国名牌的突破。“普九”水平不断提升，高中段教育普及率提高41个百分点，普通高考本科录取人数年均增长30%以上，学前教育、职业教育、成人教育、高等教育协调发展，人均受教育年限达到8.3年。可持续发展成效明显。连续五年被省委、省政府授予人口和计划生育目标管理一等奖。水、土地、海洋、森林等资源保护工作进一步加强，耕地总量保持动态平衡。全面实施生态市建设规划，积极探索发展循环经济，大气质量、饮用水水质和近岸海域水质始终保持国家一级（一类）标准。各项事业取得新进步。文化事业繁荣活跃，新闻中心、广电中心、会议中心建成启用。疾病预防控制和医疗救治“两个体系”建设不断加强，两县两区全部推行了新型农村合作医疗，区域覆盖率在全省率先达到100%。争取国家批复设立了岚山区。连续两次被授予“全国双拥模范城”称号。社会慈善、扶残助残和老龄事业协调发展，民族宗教、人防、史志、档案、气象、地震等各项工作也都取得新的进步。精神文明和民主法制建设得到加强。以建设文明城市为目标，广泛开展了多种形式的文明创建活动。圆满完成“四五”普法规划和依法治市第二个五年规划，农村民主政治建设不断深入。反腐败斗争力度进一步加大，查处了一批大案要案。“平安日照”建设扎实推进，严厉打击各类刑事犯罪，妥善处理人民内部矛盾，强化安全生产监督管理，全市社会保持和谐稳定。人民生活水平不断提高。2005年城市居民人均可支配收入达到9809元，农民人均纯收入达到4123元，年均分别增长8.6%和7.7%。新增城镇就业再就业14.8万人，城镇登记失业率控制在3.5%以内。社会保障体系进一步健全，覆盖面不断扩大，保障能力明显增强。城市低保实现动态管理下的应保尽保，农村特困群众得到有效救助。95%的村通上了柏油路，实现了村村通客车，农村生产生活条件进一步改善。

政府自身建设不断加强，行政能力有了新提高。按照“建设干事创业政府、一心为民政府、务实高效政府、公正廉洁政府”的承诺，始终把解放思想、勇于创新作为政府工作的根本动力，坚持“凡是能交给市场去办的事情，政府决不包揽；凡是能用经济手段解决的问题，决不用行政手段；凡是能够花钱直接办的事情，决不拿钱养人去办”，各级政府改革创新的力度越来越大，驾驭市场经济的能力明显增强。特别是通过设立“政府创新奖”，有效地调动了各级改革创新的积极性，三年来共有60多个单位申报创新成果343项，其中36项获“政府创新奖”。始终把敢想敢干、争创一流作为政府工作的目标追求，努力做到立说立行、干就干好，许多工作实现了重大突破。日照能从300多个城市的竞争中脱颖而出，成功入围中国“魅力城市”；能将中国电影金凤凰奖颁奖晚会和央视明星足球赛等活动首次争取到地级市来举办；能建议国家体育总局设立并成功申办首届中国水上运动会；能实现打通日照港东西港区、拓展岚山北港区的发展目标；特别是能以超常的速度开通到韩国的客箱班轮，日照钢铁厂当年签约、当年开工、当年投产，争取岚山成功设区，圆了全市人民盼望已久的“通航梦”、“钢铁梦”和“设区梦”，都是坚持敢想敢干、勇争一流的结果。始终把政务公开、依法行政作为政府工作的基本准则，以提高政府工作透明度、扩大人民群众知情权为目标，大力实施政府工作“阳光工程”，建立了政府信息公开、重大事项新闻发布会和市政府常务会议邀请新闻媒体列席制度，开通了“12345”市长公开电话和“行风在线”直播节目，在全省率先实行市长接听电话日制度，进一步畅通了政府与群众联系的渠道。认真学习贯彻《行政许可法》，不断提高各级政府公务员依法行政的能力和水平。设立了行政投诉监察中心，加强财政监督和审计工作，主动接受人大、政协监督，五年累计办理人大代表建议259件、政协委员提案760件，基本做到了件件有回音、事事有着落。

各位代表，刚刚过去的2005年，是我市坚持以科学发展观统领全局，全力加快经济社会发展的一年。初步统计，全市生产总值比上年增长17.5%，地方财政收入增长31.4%，规模以上固定资产投资增长58%，社会消费品零售总额增长15.6%，实际外商直接投资增长176.4%，进出口总额增长38.2%，城市居民人均可支配收入增长8%，农民人均纯收入增长11.6%。主要指标增幅位居全省前列，高于“十五”时期平均增长速度，为实施“十一五”规划奠定了坚实的基础。

回顾五年来的工作，我们主要把握了以下几点：一是坚定不移地贯彻执行党的路线方针政策和重大决策部署，牢牢抓住发展这个第一要务不放松。五年来，面对突如其来的非典和禽流感疫情，面对国家加强宏观调控和区域经济发展竞争激烈的态势，全市上下认真贯彻执行党中央、国务院的一系列决策部署和省委、省政府“一二三四五六”的工作思路，正确判断形势，积极有效应对，始终锁定跨越发展的目标，做到在任何时候、任何情况下都坚持以经济建设为中心不动摇，坚持以科学发展观统领经济社会发展全局，抓住用好各种机遇，全力加快经济社会发展，使全市经济运行的稳定性、协调性和可持续性进一步增强，“十五”成为建市以来我市发展最快最好的时期之一。二是坚持发挥优势，扎扎实实地做好上情与下情结合的文章。五年来，我们始终坚持从日照实际出发，创造性地贯彻落实上级指示精神，认真实施“港口立市、工业强市、科教兴市、生态建市”四大战略，狠抓招商引资、县区经济、接轨青岛、农民增收等牵动全局的重点工作，特别是充分发挥港口、区位、生态环境三大优势，大力培育特色鲜明、竞争力强的地方经济品牌，努力把自然优势转化为竞争优势、发展优势，使港口经济、旅游经济、大学经济、体育经济等特色经济成为我市经济社会发展中非常靓丽的品牌和亮点。三是坚持改革创新，不断增强经济社会发展的活力。五年来，我们坚持解放思想、改革创新，努力做到“用招商引资的钱发展经济，用市场化运作的钱建设城市，用财政的钱改善人民生活”，取得了明显成效。近年来城建投资每年都在20亿元左右，这些资金大都来自于市场化运作。在加大城建投入的同时，不仅没有增加政府贷款和群众负担，而且还偿还了历年形成的财政欠款4.4亿元。对承办的大型体育赛事、文化活动和茶博会、房博会全部实行市场化运作。用创新的理念成功举办了两次银企合作促进会，融通资金157亿元，有效地缓解了经济发展的资金瓶颈。特别是连续三年实施经济适用房货币直补改革，为1736户城市低收入家庭解决了住房困难，共发放补贴9195万元，户均补助5万多元，这一做法得到了各级领导和专家的充分肯定，被誉为“日照模式”，曾培炎副总理、张高丽书记、韩寓群省长分别作出批示，新华社、中央电视台、《人民日报》等新闻媒体和《红旗文稿》、《山东通讯》等刊物都予以报道和宣传推广。四是坚持一心为民，实现好、维护好、发展好人民群众的根本利益。始终把执政为民作为一切工作的出发点和落脚点，对关系经济社会发展全局和群众切身利益的重大问题，坚持抓住不放、一抓到底，尽心竭力地为群众办实事、做好事、解难事，努力让人民群众得到更多的实惠。坚持向社会公开征集的办法，每年确定十几件与人民群众生产生活息息相关的实事，并明确责任分工，认真组织实施，确保事事都落到实处。事实证明，只有坚持以人为本，让广大人民群众充分享受改革发展的成果，我们的工作才能够得到人民群众的支持和拥护，我们的发展才有意义，我们的事业才能够无往而不胜！五是坚持用心把握、用心工作，创造良好的发展环境。五年来，我们正确处理改革发展稳定的关系，始终坚持正确的思想导向，把干部群众的思想引导到想发展、谋发展、加快发展上来；始终坚持正确的舆论导向，着力营造风正气顺心齐、团结和谐稳定的良好氛围；始终坚持正确的工作导向，以干事创业的实绩论英雄，鼓励引导广大干部群众积极投身到改革开放和经济建设的主战场，努力做到想干、会干、干好，形成了建设日照、发展日照的强大合力。

总之，“十五”时期的五年，是我市综合经济实力明显增强，城乡面貌明显变化，人民生活水平明显提高的五年。在全市人民的共同努力下，今天的日照正以经济快速发展、社会和谐稳定、人民安居乐业、充满生机活力的崭新形象，站在了一个新的历史起点上！五年的辉煌成就来之不易，这是党中央、国务院、省委、省政府和市委正确领导的结果，是各级人大、政协和社会各界大力支持的结果，是全市人民解放思想、开拓创新、顽强拼搏的结果。在此，我代表市人民政府，向所有为日照改革和发展做出贡献的同志们、朋友们，表示崇高的敬意和衷心的感谢！

各位代表，我市经济和社会发展虽然取得了令人鼓舞的成就，但前进中还存在不少矛盾和问题。从总体上看，经济总量尚小，沿海欠发达的面貌还没有根本改变；结构层次偏低，二三产业比重仍低于全国、全省平均水平；农业产业化水平和抗御自然灾害的能力不高，农民增收的长效机制尚未建立起来；城乡之间发展不平衡，县区经济实力还比较薄弱；投资环境有待进一步改善，对外开放的水平与沿海城市的地位还不适应；城市化水平不高，城中村改造的任务相当繁重；自主创新能力不够强，科技力量薄弱、人才匮乏的问题还没有得到有效解决；社会事业发展欠账较多，社会保障能力还很脆弱，社会生活中还存在一些不稳定因素。面对纷繁复杂的形势和市场经济发展的要求，我们的思想观念还不够适应，政府职能转变还不够到位，依法行政能力需要进一步提高；一些地方、单位和政府工作人员服务意识淡薄，官僚主义、形式主义作风和腐败现象依然不同程度地存在。对以上存在的矛盾和问题，我们一定高度重视，采取有力措施，切实加以解决。

二、“十一五”时期经济社会发展的总体要求

从今年开始，已进入第十一个五年规划时期。制定一个科学的、切实可行的发展规划，对于落实科学发展观，全面建设小康社会，加快推进现代化建设，具有十分重要的意义。按照中央和省、市委的部署要求，市政府编制了《日照市国民经济和社会发展第十一个五年规划纲要（草案）》。《纲要（草案）》及《说明》已印发大会，请一并予以审议。

《纲要（草案）》确定“十一五”时期我市经济社会发展的指导思想是：以邓小平理论和“三个代表”重要思想为指导，深入贯彻落实党的十六大和十六届三中、四中、五中全会精神，以科学发展观统领全局，紧紧围绕又快又好发展这个根本要求，着力抓好改革创新和结构调整两个关键，深入实施港口立市、工业强市、科教兴市、生态建市战略，加快工业化、城市化、市场化、国际化进程，全面推进和谐社会建设，努力赶超全省平均水平，为提前建成全面小康社会、提前基本实现现代化奠定坚实基础。“十一五”时期经济社会发展的主要目标确定为：

——经济实力显著增强。到2010年主要经济指标在2005年的基础上再翻一番，全市生产总值突破1000亿元，年均增长15%，人均达到34200元；地方财政收入达到29.7亿元，年均增长15%；三次产业比例调整为8:57:35；社会消费品零售总额达到245亿元，年均增长15%。

——经济增长方式明显转变。循环经济进一步发展，资源利用效率明显提高，万元GDP综合能耗比“十五”末降低20%，工业用水重复利用率达到80%。生态市建设步伐加快，城市生活垃圾无害化处理率达到100%，城市生活污水处理率提高到90%，森林覆盖率达到35%。科技创新能力得到明显增强，社会研发投入占GDP比重达到2%，高新技术产业产值占规模以上工业总产值的比重达到20%。

——社会主义市场经济体制不断完善。投资、财税、卫生、社会保障、公共管理体制改革取得突破性进展，体制机制创新能力显著增强，市场配置资源的基础性作用得到充分发挥，统一、开放、竞争、有序的市场体系基本形成。开放型经济达到新水平，进出口总值年均增长20%，实际外商直接投资年均增长30%。

——城乡发展更加协调。县区经济实力明显增强，发展环境更加优化，城乡基础设施更加完善，城市化水平达到50%。农村各项改革全面深化，农村公共事业建设得到加强，社会主义新农村建设步伐加快，城乡一体化发展格局初步形成。

——人民生活水平显著改善。城乡居民收入进一步增加，消费结构日趋合理，生活质量不断提高。城市居民人均可支配收入达到14400元，农民人均纯收入达到5800元，年均分别增长7%左右。各项社会事业进一步发展，居住、交通、教育、文化、卫生和环境等方面的条件有较大改善，人均受教育年限达到9年以上，万人拥有医生数和病床数超过13人和24张，人口自然增长率控制在5.8‰。

——社会和谐程度继续提高。就业政策得到全面落实，城乡一体化的劳动力市场不断完善，城镇居民相对充分就业的目标基本实现，每年新增城镇就业3.85万人，新增跨区域转移就业2万人，城镇登记失业率控制在3.5%以内。社会保险、社会救助、社会福利与慈善事业相衔接的社会保障体系更加完善。民主法治建设和精神文明建设取得新进展，社会治安和安全生产状况进一步好转，和谐日照建设取得新进步。

做好“十一五”期间的工作，实现上述任务目标，必须坚持以科学发展观统领经济社会发展全局，努力做到“五个更加注重”。一是更加注重科学发展，按照全面协调可持续发展的要求，转变发展观念、开拓发展思路、创新发展模式、提高发展质量，真正自觉地把科学发展观贯彻于“十一五”始终，落实到经济社会的各个环节，努力保持经济又快又好地发展。二是更加注重结构调整，继续按照“优化提高第一产业，突出发展第二产业，繁荣发展第三产业”的思路，抓好产业结构战略性调整，着力转变经济增长方式，加快经济结构优化升级。三是更加注重城乡协调，发展壮大县区经济、乡镇经济，加快建设社会主义新农村，努力实现共同富裕。四是更加注重改革开放和科技创新，充分发挥市场配置资源的基础性作用，进一步提高对外开放水平和技术创新水平，不断增创发展新优势。五是更加注重社会和谐，坚持以人为本，从最广大人民的根本利益出发谋发展、搞建设，努力促进社会主义物质文明、政治文明、精神文明、社会文明协调发展。

各位代表，“十一五”是我市加快推进现代化建设的关键时期。这五年将是日照经济站在新起点、实现新发展，努力赶超全省平均水平的五年；将是城市建设内涵提高，国际性滨海城市特色不断显现的五年；将是全市人民享受更多的发展成果、生活水平明显提高，整个社会日益和谐的五年。纵观这一时期的国内外环境，总体上看机遇与挑战并存、机遇大于挑战，是可以大有作为的战略机遇期。特别是中央确立了科学发展观的指导方针，为我们指明了前进的方向；我市得天独厚的发展优势和建市以来打下的物质基础，为我们实现又快又好发展创造了有利的条件；全市上下解放思想、干事创业、勇争一流的氛围更加浓厚，为我们加快发展提供了强大的动力。只要我们牢牢把握重要战略机遇期，充分利用好这些有利条件，进一步凝聚起全市上下的智慧和力量，坚定信心，开拓进取，真抓实干，我们的奋斗目标就一定能够实现，日照的明天一定会更好！

三、2006年的目标任务和工作重点

2006年是实施“十一五”规划的开局年，是站在新起点实现新发展的起步之年。我们要坚持以邓小平理论和“三个代表”重要思想为指导，认真贯彻党的十六届五中全会和中央、省市经济工作会议精神，以科学发展观统领全局，着眼长远、突破重点、统筹兼顾、科学发展，努力为“十一五”开好局、起好步。主要预期目标是：生产总值增长16%，规模以上工业增加值增长25%，固定资产投资增长30%，社会消费品零售总额增长15%，进出口总额增长20%，地方财政收入增长15%，城市居民人均可支配收入增长8%，农民人均纯收入增长7%，城镇登记失业率控制在3.2%，人口自然增长率控制在6.9‰。今年要着重抓好以下七个方面的工作：

（一）统筹城乡发展，扎实推进社会主义新农村建设。按照“生产发展、生活宽裕、乡风文明、村容整洁、管理民主”的要求，有计划、有步骤、有重点地加快推进社会主义新农村建设，力争今年迈出坚实的步伐。

一是大力发展农村经济，促进农民增收。在稳定粮食生产的基础上，继续调整优化农业内部结构，引导农民发展特色高效农业，做大做强畜牧业和水产业，挖掘农民增收的潜力；以培植农业龙头企业为重点，加快农业产业化进程，积极发展农村二三产业，扩大农民增收的空间；坚持走“减少农民、富裕农民”的路子，全力促进农村劳动力有序转移，拓展农民增收的渠道。同时，在“多予、放活”上下功夫，全面落实支农惠农政策，年内全部取消农业税，进一步减轻农民负担。

二是发展壮大县区经济，增强辐射带动能力。加快发展县区经济是建设社会主义新农村的关键所在。要继续把工业化作为加快县区经济发展的突破口，将两县纳入港口腹地，鼓励扶持县区依托港口优势，因地制宜，尽快把工业经济做大做强。继续把外向化作为加快县区经济发展的目标取向，依托资源、环境优势，加大招商引资力度，培植强势产业。继续把城市化作为加快县区经济发展的重要依托，按照高起点规划、高水平建设、高效能管理的要求，加快县城、中心城镇的建设和发展。搞好园区和加工基地建设，按照经济发展区域化的要求，鼓励发展“飞地经济”，引导企业向园区集中。加快经济强镇的建设步伐，带动促进乡镇经济的快速发展。

三是加强农业和农村基础设施建设，努力改善农村面貌。坚持资金投入向农村倾斜，基础设施向农村延伸，公共服务向农村覆盖，切实加大农村“路水电医学”等方面的投入，集中财力为农民群众办几件看得见、摸得着的实事。在去年基本实现农村公路“村村通”的基础上，从今年开始省市合计每年安排2400万元，启动村村通自来水工程，力争用三年时间基本完成。安排1200万元用于新型农村合作医疗补助。投资425万元改造乡镇卫生院。投资1000万元用于农村中小学危房改造。同时，分期分批地搞好乡镇敬老院的改造建设。加快有线电视“村村通”工程，大力发展农村文化事业，切实提高农民生活质量。

（二）全力加快工业化进程，不断提高制造业水平。坚持把发展工业作为经济工作的重中之重，坚定不移地实施“工业强市”战略，继续抓好“三个一批”，加快推进工业化进程。

一是立足港口优势，培育壮大一批支柱产业。以培植钢铁、能源、浆纸、化工、机械、食品六大临港产业和建材、纺织服装、木制品加工等传统产业为重点，坚持“大项目—产业链—产业基地”的发展方向，争上一批投资规模大、带动能力强的大项目，并依托这些项目，大力发展上下游产品和配套企业，拉长产业链条，培育产业集群，形成集聚效应。经过三五年的努力，力争把日照打造成为全国重要的钢铁生产基地、化工基地、能源基地、浆纸生产基地、粮油加工基地和机械制造业基地。

二是坚持引进培植并重，着力发展一批大企业集团。大企业是带动区域经济发展和增加财政收入的重要力量。坚持一手抓引进、一手抓培植，引进建成一批能够加速经济发展、促进结构优化的大项目。加大对50户重点企业的培育力度，促其快速膨胀、健康发展。年内日照钢铁销售收入确保过百亿元，结束我市无销售收入过百亿元企业的历史。与此同时，通过招商引资、自主创新和成果转化等各种途径，加快培植一批科技型企业集团，尽快扩大高新技术产业规模，力争全市高新技术产业产值增长50%以上。

三是实行激励扶持政策，积极培育一批知名品牌。坚持把争创名牌与培育品牌企业、培植支柱产业结合起来，综合运用科技、经济、法律、行政等手段，积极培育名牌产品，努力以名牌产品做大品牌企业，以品牌企业带动产业发展。坚持对名牌产品实行奖励的政策，充分调动企业争创名牌的积极性，切实提高企业核心竞争力，推动全市工业快速发展。

（三）大力培育特色经济，繁荣发展服务业。发达的服务业，是现代经济的重要标志。要充分发挥我市港口、区位、生态环境优势，大力发展服务业，努力使我市服务业在规模和水平上有新的突破。

一是大力发展以港口为重点的物流业。按照建设两个亿吨大港的目标，加大港口投入，进一步完善港口功能，增强港口竞争力。年内完成港口投资37亿元，着力抓好日照港35万吨级矿石码头、中港区回填工程、30万吨级原油码头等重点工程建设。大力开拓港口腹地和市场，强化港路、港企合作，确保年内港口货物吞吐量过亿吨，使日照港跨入亿吨大港的行列。进一步优化港口发展环境，启动口岸通关中心和电子口岸建设。规划建设港口物流园区和保税物流中心，加快推进南方家园物流商城等商贸设施建设，积极培育以第三方物流和专业物流园区为重点的现代物流业。

二是加快发展旅游经济、体育经济、大学经济、会展经济等特色服务业。进一步修编完善《日照市旅游发展规划》和山海天旅游度假区总体规划，加快推进太阳城主题公园等项目的规划建设，提升景区景点和旅游资源开发的层次与水平，年内力争实现旅游收入40亿元。办好国际470级帆船世界锦标赛等重大体育赛事，精心做好2007年首届中国水上运动会筹备工作，积极开发体育旅游、休闲健身项目，大力发展体育产业。进一步规范和促进房地产业健康发展。加快大学科技园建设步伐，搞好山东外国语职业学院、清大海诺科技孵化中心等项目建设。采取市场化运作的办法，着手规划建设具有国际一流水准的会展中心，促进会展经济发展。

三是积极发展金融、信息、中介、社区服务等现代服务业。大力推进“诚信日照”建设，优化金融生态环境，广泛吸引外来金融机构在日照发展业务，实现金融与经济发展良性互动、互利共赢。搞好政府门户网站和政务专网平台建设，完善社会信息服务网络，全力提高我市的信息化水平。大力培育发展各类中介服务业，为企业经营、人民生活提供有效的社会化服务。加强社区基础设施建设，推进社区服务产业化进程。与此同时，按照“创特色、上水平”和“规范管理、便民利民”的要求，改造提升商贸流通、餐饮娱乐等传统服务业，努力做活早市、做旺午市、繁荣夜市，提高城市繁华程度。大力推进“万村千乡”市场工程，引导连锁店和超市加快向农村延伸发展。整顿和规范市场秩序，加强市场监管，促进服务业健康发展。

（四）着眼于建设国际水准的滨海城市，进一步提高城市规划建设管理水平。坚持用高水平规划引领城市建设。按照“双城一区、轴向拓展”的带状城市发展方向，高起点、高标准完成城市总体规划修编，进一步优化城市空间布局。完成城市交通体系等专业规划、岚山分区规划及重点区域的控制性详规，力争详规覆盖率达到90%。进一步放开规划设计市场，提升规划设计水平，努力做到用高水平的规划指导城市建设。坚持以项目为抓手推动城市建设。突出抓好三类项目：一是彰显城市特色的精品项目。集中力量搞好投资14亿元的奥林匹克水上公园建设，配套完善灯塔广场、世帆赛基地，努力将奥林匹克水上公园建成能够展现我市“水上运动之都”和国际水准滨海城市形象的精品工程。二是提升城市功能的基础项目。年内完成投资4.5亿元的滨海大道建设工程，贯通北京路、西安路等城区道路，推进日照至潍坊、岚山至枣庄高速公路建设的进程。加快市垃圾处理厂、污水处理厂二期工程建设，完善城市供水、供气、供热、治污设施，进一步强化城市载体功能。着眼于筹办大型体育赛事和会展活动，加快建设建国君豪、新天地等四星级以上酒店，提升城市服务水平。三是便民惠民的服务项目。修建改造一批便民道路，抓好一批绿化美化亮化项目的建设，搞好沙墩河等河道治理，加快城中村改造步伐，切实改善市民生产生活环境。坚持建设管理并重提升城市建设。进一步巩固“双创”成果，继续深入开展城市环境综合整治，加大城市管理综合执法力度，加强城市交通、市容环境和卫生管理，着力把城市管理提升到一个新水平。

（五）进一步深化改革，全方位扩大对外开放。坚持把市场化取向作为改革的主题，紧紧抓住影响经济社会发展的突出矛盾和问题，更大程度地发挥市场配置资源的基础性作用。一是以转变政府职能为重点推进行政管理体制改革。进一步理顺政府与企事业单位、市场中介组织的关系，精简规范行政审批事项，强化社会管理和公共服务职能，切实解决好政府职能错位、越位、缺位的问题。积极推进乡镇机构改革，精简机构和减少财政供养人员，切实减轻财政和农民负担，确保乡镇基层组织正常运转。二是以事改企、企改制为重点推进事业单位改革。坚持分类管理，建立政事职责分开、法人地位明确、单位自主运营、政府依法管理的事业单位管理体制和运行机制。对主要从事生产经营和开发服务的事业单位，原则上都要改企转制。全面推行事业单位法人负责制和人员聘用制，建立有利于优秀人才脱颖而出、充满生机与活力的用人制度和分配制度。三是以推进公共财政体系建设为重点深化财税体制改革。切实搞好部门预算、国库集中支付和政府采购等项改革，规范非税收入和国有资产管理，全面推行“金财工程”。四是以改善环境为重点促进非公有制经济发展。着力营造平等竞争的法制环境、政策环境和市场环境，鼓励、支持非公有制经济加快发展。进一步放宽市场准入，推动非公有资本进入法律法规未禁止的各个领域。

对外开放是加快日照发展的根本出路。要继续坚定不移地实施经济国际化战略，全面提高对外开放水平。一是积极有效地利用外资。紧紧抓住国内外产业转移的机遇，创新招商方式，加大招商力度，拓展招商领域，实现招商引资工作的新突破。全年实际外商直接投资超过2亿美元，利用内资突破300亿元。坚持把改善投资环境作为扩大招商引资的关键措施，进一步完善基础设施硬环境，重点抓好日照经济开发区、高科技工业园、岚山经济开发区和各区县工业园的配套建设。同时，下大力气抓好软环境建设，在全社会营造亲商、安商、富商的浓厚氛围。二是加快转变外贸增长方式。调整优化出口产品结构，引导出口企业从数量型向质量效益型转变。坚持把加工贸易作为发展外向型经济的突破口，积极争取建立国家级出口加工区，尽快把我市建设成外向型的加工基地、装配基地、出口基地。加快发展对外经济技术合作，鼓励有条件的企业到国外投资办厂、开展工程承包和劳务输出，促进对外经贸的持续快速发展。三是加大接轨青岛融入半岛力度。继续坚持“观念接轨为先导、产业接轨为核心、企业接轨为主体”的思路，增强主动意识，加大工作力度，进一步拓宽和提升与青岛等半岛城市交流合作的领域和水平，努力在务实合作上取得新的突破。

（六）深入实施“科教兴市”战略，提高自主创新能力。全面落实全国科学技术大会精神，努力把我市建设成创新型城市。一是加快推进科技进步。坚持自主创新、重点跨越、支撑发展、引领未来的指导方针和有所为、有所不为的原则，以市场为导向，以企业为主体，以提高自主创新和成果转化能力为重点，突出抓好科技成果引进转化、高新技术产业培育、技术创新体系建设，提升产业层次和技术水平，切实增强经济核心竞争力。深化科技体制改革，加大科技经费投入，今年科技应用技术研究开发经费投入确保增长60%以上。二是加快教育事业发展。进一步优化教育资源配置，完善以县为主的义务教育体制，认真落实“两免一补”政策，高中段教育普及率要提高到87%。大力发展高等教育、职业教育、成人教育，年内创办日照市高级技工学校。深化办学体制改革，鼓励社会力量办学，实现教育投入多元化。三是加强人才队伍建设。坚持“用好现有的、引进急需的、培养未来的”方针，全面实施人才强市战略，加强创新型人才培养，抓好党政领导人才、企业经营管理人才、专业技术人才三支队伍建设，为经济社会发展提供强有力的智力支持和人才支撑。

（七）建设文明平安日照，促进社会和谐发展。构建社会主义和谐社会，是经济社会发展的重要目标，也是推进经济社会发展的重要保障。按照民主法治、公平正义、诚信友爱、充满活力、安定有序、人与自然和谐相处的要求，加快和谐社会建设。一是积极建设环境友好型资源节约型社会。按照“节约发展、清洁发展、安全发展”的要求，大力发展循环经济，切实抓好循环经济型企业、生态工业园、生态社区和环境优美乡镇的建设，加强对水源、土地、森林、海洋等自然资源的生态保护，严格落实“三同时”和一票否决制，加大污染防治力度，保持好蓝天、碧海、金沙滩的环境优势。要以建设节约型社会为目标，积极推进节能节水节地节材，严格限制高耗能、高耗材和高耗水行业发展，大力倡导节约型发展模式。二是加强就业和社会保障工作。把促进就业放在优先位置，切实搞好对困难群体的再就业援助，改善对进城务工人员的就业服务，努力形成市场主导、城乡统筹和多渠道灵活就业的格局。完善社会保障体系，扩大养老、医疗、失业、工伤等社会基本保险覆盖面，逐步做实企业养老保险个人帐户。建立完善城乡居民最低生活保障制度和失地农民基本养老保险制度。认真落实优抚政策，支持社会慈善、社会捐赠、群众互助等社会救助活动。年内建立起廉租住房制度，对住房困难的城市最低收入家庭发放廉租住房补贴，确保弱势群体能够享受到更多的温暖与实惠。三是全面推进“平安日照”建设。正确处理新形势下的人民内部矛盾，重视群众来信来访，积极做好矛盾纠纷排查调处。加强社会治安综合治理，抓好治安防控体系建设，严厉打击严重危害人民群众生命财产安全的犯罪活动，切实增强人民群众的安全感。强化“一岗双责”安全生产责任制，逐步建立安全生产长效机制，加强食品、药品监管，保障人民群众生命安全。健全完善应急预案体系，提高保障公共安全和处置突发事件的能力。四是加强民主法制建设。进一步完善基层民主，大力推行村务公开、厂务公开、政务公开。加强司法监督，维护司法公正。推进依法治市，深入开展普法宣传教育，做好法律服务和法律援助工作。各级政府都要支持审判机关和检察机关依法独立行使审判权和检察权。五是统筹发展各项事业。深入开展群众性精神文明创建活动，提高全民思想道德素质。要加强文化建设，大力发展文化产业，抓好莒县博物馆等一批重点文化设施建设，扩大有线电视和数字电视网络覆盖面。加强疾病预防控制体系、医疗救治体系、应急指挥体系和公共卫生预警体系建设，整顿和规范医疗服务秩序。做好人口和计划生育工作，稳定低生育水平，提高人口素质。积极开展全民健身运动，关心支持妇女儿童、残疾人、老年人服务事业。抓好全民国防教育和国防后备力量建设，努力实现“全国双拥模范城”三连冠。以贯彻落实省地方史志工作条例为契机，扎实推进依法修志。继续做好禽流感防控、档案、人防、气象信息服务等各项工作，促进经济社会协调发展。

四、切实加强政府自身建设

实现“十一五”宏伟目标，各级政府重任在肩，责无旁贷。我们一定按照胡锦涛总书记提出的“为民、务实、清廉”的要求，继续履行“建设干事创业政府，做到创业有为；建设一心为民政府，做到为民有绩；建设务实高效政府，做到工作有效；建设公正廉洁政府，做到廉政有方”的郑重承诺，不断加强政府自身建设，全力开创政府工作的新局面。

始终把发展作为第一要务，坚持干事创业不动摇。要把加快发展放在首位，以振兴日照为己任，切实做到聚精会神搞建设、一心一意谋发展。牢固树立和认真落实科学发展观，推动经济社会切实转入科学发展的轨道。坚持解放思想、实事求是、与时俱进，从理论和实践的结合上不断开拓新思路、取得新突破、创建新业绩。要加强执政能力建设，切实提高政府工作人员的素质和干事创业的能力，实现日照经济社会更快更好的发展。

牢固树立公仆意识，坚持一心为民不动摇。按照以人为本、执政为民的要求，坚持“发展为了人民、发展依靠人民、发展成果由人民共享”，尽最大努力解决群众最关心、最直接、最现实的利益问题。建立健全深入了解民情、充分反映民意、广泛集中民智、切实珍惜民力的决策机制，把为民办实事作为政府工作的一项制度长期坚持下去，继续实行重大决策听证制度、公众参与制度和政务公开制度，使政府工作更加顺民心、合民意。要进一步转变工作作风，大兴求真务实之风，坚决克服官僚主义和形式主义，多到基层去，多到群众中去，进一步凝聚起广大人民群众的力量，共同推进全面建设小康社会的伟大事业。

加快转变行政职能，坚持务实高效不动摇。按照建设创新型城市的要求，深入开展政府工作创新活动，全力加快市场化取向的改革，努力做到以改革促发展、提效率。积极推进政企分开、政事分开，政府该管的事要坚决管住管好，不该管的事要坚决放开放活，使政府职能逐步转到“经济调节、市场监管、社会管理和公共服务”上来。进一步完善政府工作制度和运作机制。大力推进政府决策的科学化、民主化，健全决策目标体系；大力推进执行责任的具体化、明确化，健全执行责任体系；大力推进考核监督的制度化、规范化，健全考核监督体系，切实提高行政效率和服务水平。要围绕人代会确定的目标任务，用心尽力地抓好工作落实，确保抓出效果、干出水平。

严格依法行政，坚持廉洁勤政不动摇。高度重视法制建设，每一位政府工作人员无论职务多高、权力多大，都要时刻绷紧依法行政这根弦，一切行政行为都要遵循法律法规，始终牢记有权必有责、用权受监督、违法要追究、侵权要赔偿。要坚持向人大及其常委会报告工作和向人民政协通报情况的制度，自觉接受人大及其常委会和人民政协的监督，高度重视人大代表、政协委员的建议、提案办理工作。认真听取民主党派、工商联、无党派人士和各人民团体的意见。要进一步加强各级政府的廉政建设，建立健全教育、制度、监督、惩处并重的行之有效的反腐败体系，扎实推进反腐倡廉工作，切实纠正部门和行业不正之风，始终保持为民、务实、清廉的良好形象。

各位代表，日照的改革和发展正站在新的历史起点，新的形势催人奋进，新的征程任重道远。让我们更加紧密地团结在以胡锦涛同志为总书记的党中央周围，高举邓小平理论和“三个代表”重要思想伟大旗帜，全面贯彻落实科学发展观，在中共日照市委的领导下，团结拼搏，开拓前进，奋力开创经济社会又快又好发展的新局面，为圆满完成全年各项工作任务和五年的发展目标，加快全面建设小康社会进程而努力奋斗！
